# Tryptophan Metabolites, Cytokines, and Fatty Acid Binding Protein 2 in Myalgic Encephalomyelitis/Chronic Fatigue Syndrome

**DOI:** 10.3390/biomedicines9111724

**Published:** 2021-11-19

**Authors:** Manuela Simonato, Stefano Dall’Acqua, Caterina Zilli, Stefania Sut, Romano Tenconi, Nicoletta Gallo, Paolo Sfriso, Leonardo Sartori, Francesco Cavallin, Ugo Fiocco, Paola Cogo, Paolo Agostinis, Anna Aldovini, Daniela Bruttomesso, Renzo Marcolongo, Stefano Comai, Aldo Baritussio

**Affiliations:** 1 PCare Laboratory, Fondazione Istituto di Ricerca Pediatrica, Citta’ della Speranza, 35127 Padova, Italy; m.simonato@irpcds.org; 2 Department of Pharmaceutical and Pharmacological Sciences, University of Padua, 35131 Padua, Italy; stefano.dallacqua@unipd.it (S.D.); stefania.sut@unipd.it (S.S.); 3 Pediatrician, Via Galvani 6, 35020 Padua, Italy; zillicat@gmail.com; 4 Department of Medicine, University of Padova, 35128 Padova, Italy; romano.tenconi@unipd.it (R.T.); paolo.sfriso@unipd.it (P.S.); leonardo.sartori@unipd.it (L.S.); ugo.fiocco@unipd.it (U.F.); daniela.bruttomesso@unipd.it (D.B.); renzo.marcolongo@aopd.veneto.it (R.M.); aldo.baritussio@unipd.it (A.B.); 5 Department of Laboratory Medicine, Policlinico Azienda Ospedaliera di Padova, 35128 Padova, Italy; nicoletta.gallo@aopd.veneto.it; 6 Independent Statistician, 36020 Solagna, Italy; cescocava@libero.it; 7 Department of Medicine, University Hospital Santa Maria della Misericordia, University of Udine, 33100 Udine, Italy; paola.cogo@uniud.it; 8 Department of Medicine, Ospedale Sant’Antonio Abate, Azienda Sanitaria del Friuli Centrale, 33100 Udine, Italy; agostinispaolo@gmail.com; 9 Department of Medicine, Boston Children’s Hospital, Boston, MA 02115, USA; anna.aldovini@childrens.harvard.edu; 10 Department of Pediatrics, Harvard Medical School, Boston, MA 02115, USA; 11 Department of Biomedical Sciences, University of Padua, 35121 Padua, Italy; 12 Department of Psychiatry, McGill University, Montreal, QC H4H 1R3, Canada; 13 Division of Neuroscience, IRCSS San Raffaele Scientific Institute, 20132 Milan, Italy

**Keywords:** ME/CFS heterogeneity, cytokines, intestinal permeability, tryptophan metabolism, kynurenine pathway, 3-hydroxykynurenine, kynurenine, serotonin, biomarkers, personalized medicine

## Abstract

Patients with Myalgic Encephalomyelitis/Chronic Fatigue Syndrome (ME/CFS) differ for triggers, mode of start, associated symptoms, evolution, and biochemical traits. Therefore, serious attempts are underway to partition them into subgroups useful for a personalized medicine approach to the disease. Here, we investigated clinical and biochemical traits in 40 ME/CFS patients and 40 sex- and age-matched healthy controls. Particularly, we analyzed serum levels of some cytokines, Fatty Acid Binding Protein 2 (FAPB-2), tryptophan, and some of its metabolites via serotonin and kynurenine. ME/CFS patients were heterogeneous for genetic background, trigger, start mode, symptoms, and evolution. ME/CFS patients had higher levels of IL-17A (*p* = 0.018), FABP-2 (*p* = 0.002), and 3-hydroxykynurenine (*p* = 0.037) and lower levels of kynurenine (*p* = 0.012) and serotonin (*p* = 0.045) than controls. Changes in kynurenine and 3-hydroxykynurenine were associated with increased kynurenic acid/kynurenine and 3-hydroxykynurenine/kynurenine ratios, indirect measures of kynurenine aminotransferases and kynurenine 3-monooxygenase enzymatic activities, respectively. No correlation was found among cytokines, FABP-2, and tryptophan metabolites, suggesting that inflammation, anomalies of the intestinal barrier, and changes of tryptophan metabolism may be independently associated with the pathogenesis of the disease. Interestingly, patients with the start of the disease after infection showed lower levels of kynurenine (*p* = 0.034) than those not starting after an infection. Changes in tryptophan metabolites and increased IL-17A levels in ME/CFS could both be compatible with anomalies in the sphere of energy metabolism. Overall, clinical traits together with serum biomarkers related to inflammation, intestine function, and tryptophan metabolism deserve to be further considered for the development of personalized medicine strategies for ME/CFS.

## 1. Introduction

Myalgic Encephalomyelitis/Chronic Fatigue Syndrome (ME/CFS) is a multisystem condition characterized by chronic fatigue, post-exertional malaise, unrefreshing sleep, cognitive changes, autonomic disturbances, flu-like symptoms, abdominal complaints, and intolerance to stress, noise, light, heat, or cold. Females are more frequently affected, and over half of cases happen after infection [1].

Several pathogenetic mechanisms have been proposed. Some disease manifestations, such as flu-like symptoms, indicate an inflammatory/immune basis [2]. An immune basis for ME/CFS is also suggested by evidence showing increased cytokine levels in plasma and cerebrospinal fluid and T-cell dysfunction and by imaging data pointing to an involvement of the microglia [3,4]. Furthermore, studies on the intestinal microbiome have found loss of microbial diversity and signs of increased bacterial translocation across the intestinal barrier that could also contribute to systemic inflammation [5,6].

Other disease manifestations like low exercise tolerance or the propensity to produce an excess of lactic acid during exercise hint at a metabolic basis for ME/CFS, as is also suggested by the presence of mitochondrial abnormalities [7,8].

Joining the realm of immunity with that of energy production, it has been recently shown that subsets of T cells isolated from patients with ME/CFS are unable to redirect energy metabolism towards aerobic glycolysis during activation [9]. Along the same line, poor performance during exercise could be linked to sub-optimal venous return caused by immune-mediated autonomic dis-regulation of small vessels [10].

A large body of research, based on neuroimaging, has addressed brain changes in patients with ME/CFS. The most consistent findings have been the recruitment of additional brain regions during cognitive tasks, brain stem anomalies suggestive of inflammation, and a reduction of fluorodeoxyglucose uptake, indicative of hypometabolism (reviewed by Shan et al. [11]). Brain serotonin status has also been considered, but results have been discordant, with some studies demonstrating activation of this system [3,12], while others indicate inhibition [13]. Serum levels of tryptophan, precursor and direct determinant of brain serotonin [14], have also been studied, but the results are again contrasting with some authors showing higher levels in ME/CFS [15], while others found no change [16]. Interest in tryptophan metabolism in ME/CFS is justified by the fact that tryptophan metabolites along both the serotonin and the kynurenine pathways have a role in depression [14], sleep regulation [17] and Irritable Bowel Syndrome (IBS), frequent comorbidities in patients with ME/CFS [18]. Measurements of tryptophan to kynurenine metabolites and their ratios seem thus promising in the current growing interest of developing clinically useful biomarkers of several diseases [14,19].

Patients diagnosed with ME/CFS, while displaying a platform of shared symptoms, may differ for triggers, mode of start, associated symptoms, disease evolution, and biochemical traits [15,16,20,21]. For this reason, attempts are underway to partition them into different phenotypes, most recently looking at metabolic peculiarities [22], the first stage of developing possible personalized strategies for treatment.

The scope of this work was to study clinical and biochemical traits of a cohort of patients from North-East Italy and to compare them with a group of healthy volunteers. In detail, we examined clinical data and measured serum levels of some cytokines, of a group of tryptophan metabolites via serotonin and kynurenine and of an index of altered intestinal permeability in 40 patients with ME/CFS and in 40 sex- and age-matched healthy controls. ME/CFS is emerging as a significant health issue worldwide, with an estimated prevalence of 0.8% [23], undefined pathogenesis, and no acknowledged treatment. We thought that combining clinical information with the measurement of variables reflecting different pathogenetic mechanisms could help to identify subgroups and facilitate a personalized approach to patient care.

## 2. Materials and Methods

### 2.1. Patients

From 1 January 2017 to 31 June 2018, 70 consecutive patients with unexplained fatigue were seen at the site of a charitable health care center near Padova (Italy) (Table S1). At visit 1, information was collected about diseases in the family, medical history, medication, menarche, menstrual cycle, bowel habits, smoking, alcohol consumption, use of contraceptives, duration of illness, and level of physical activity using the Bell scale [24]. Fatigue and associated symptoms were then investigated using the Canadian Clinical Criteria [25], and patients were asked to grade symptom intensity on a scale from 0 (no symptom) to 6 (maximum intensity). Patients received a physical examination. The evidence thus obtained was discussed by an internist, a pediatrician, and a geneticist, and a decision was made about the diagnosis of ME/CFS, following accepted exclusion criteria [1]. Forty-five patients were diagnosed with ME/CFS.

Afterward, contact with patients was maintained through periodic visits and, after the start of the COVID-19 pandemic, through phone calls or e-mail.

### 2.2. Case-Control Study

Patients were asked to take part in an investigation with the scope of studying serum levels of some cytokines, tryptophan metabolites, and a marker of increased intestinal permeability in ME/CFS. Forty patients were accepted. Of the five who did not, a 67-year-old female, with 22 years of disease duration, died of pancreatic cancer during recruitment, one patient declined to participate, and 3 patients could not be contacted. Forty healthy controls, matched for sex and age (26 females and 14 males; median age 33 years with interquartile range 22–46 years), were recruited among patient’s friends, school companions, medical students, and other hospital personnel. Inclusion criteria for the healthy control group were the absence of any current or past psychiatric, neurological, or other known medical conditions. Informed consent was obtained from patients and controls, and approval for the study was obtained from the Ethics Committee of Policlinico Universitario, Padova, Italy (Protocol Number 4776/AO/19). Experiments were conducted in accordance with the Declaration of Helsinki.

Patient blood samples, collected after an overnight fast, were allowed to clot at room temperature for 45 min and then were centrifuged for 10 min at 3500× *g*. Serum was aliquoted and stored at −80 °C. Aliquots were used just once after thawing. Blood samples from healthy controls were obtained within 2 weeks of the respective patient and processed in the same way. Blood collection was terminated by 1 December 2019.

### 2.3. Follow-Up

After blood sample collection, contact with patients was maintained through periodic visits and, after the start of the COVID-19 pandemic, through phone calls or e-mail. During May 2021, patients were requested to answer a questionnaire with both multiple choice and open questions concerning their feelings about their current condition and prevailing symptoms. When needed, patients were also contacted by phone. During 2021, we also checked by phone the health status of the controls used in this study.

### 2.4. Biochemical Assays

Serum cytokines were measured with ELISA. To obviate the variability between lots, sera from patients and controls were tested using the same kit [2]. ELISA kits were from Thermo Scientific, Monza, Italy (IL-4, IL-10, IL-17A, IL-18, IFN-γ) and RayBiotech, Peachtree Corner, GA, USA (IL-18). The above cytokines were chosen based on existing evidence and on the fact that for all patients’ serum levels of IL-1α, IL-1β, IL-2, IL2R, IL-6, IL-8, TNF-α, and TGF1β had already been measured at the EU-certified Central Laboratory of Padova University Hospital.

Fatty Acid Binding Protein 2 (FABP-2), an index of increased intestinal permeability [5], was measured by ELISA (myBioSource, Dan Diego, CA, USA).

**Figure 1:**
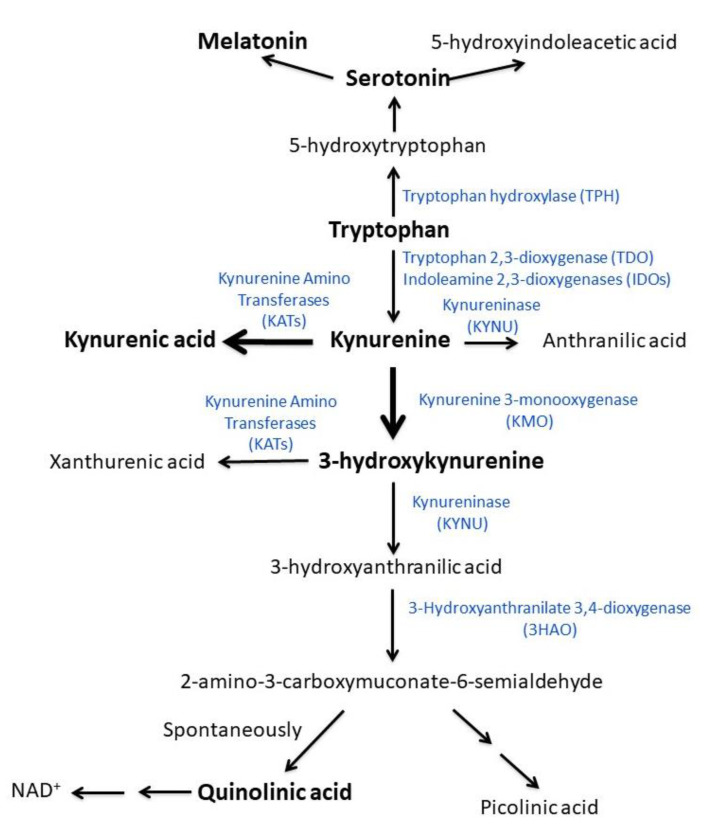
Schematic representation of the metabolism of tryptophan through the serotonin and kynurenine pathways. Enzymes involved in the different biochemical steps are indicated next to the arrow. Metabolites analyzed in this study are in bold.

The serum concentration of tryptophan and tryptophan metabolites pertaining to the Kynurenine Pathway (kynurenine, 3-hydroxykynurenine, kynurenic acid, quinolinic acid) and the Serotonin Pathway (serotonin and melatonin) were studied (Figure 1). Tryptophan, serotonin, and kynurenine were determined using a standard method in the lab [26,27] consisting of an HPLC system coupled with fluorometric and UV-Vis detectors. 3-hydroxykynurenine, kynurenic acid, quinolinic acid, and melatonin were quantified by LC-MS/MS on a Varian system composed of a binary Prostar pump, 410 autosampler, and MS320 triple quadrupole mass spectrometer equipped with Electro Spray ion source. The instrument was operating in multiple reaction monitoring modes, working in positive ion mode except for the quinolinic acid that was analyzed in negative mode. LC analysis was performed using an Agilent Eclipse XDB C8 column (3 × 150 mm, 3.5 μm) and a gradient elution with (A) water 1% formic acid and (B) Acetonitrile (0 min: 95% A; 5 min: 30% A; 8.3 min: 10% A; 10 min: 10% A; 11 min: 95% A; 15 min: 95% A) at a flow rate of 400 μL/min. The quantification of the kynurenines was computed using alfa-methyl tryptophan as an internal standard. The following ratios were used as indirect indexes of the activity of the enzymes involved in the different metabolic steps of the kynurenine pathway: kynurenine/tryptophan as an index of tryptophan 2,3-dioxygenase (TDO) and indoleamine 2,3-dioxygenase (IDO) activity; 3-hydroxykynurenine/kynurenine as an index of kynurenine 3-monooxygenase (KMO) activity; kynurenic acid/3-hydroxykynurenine as an index of the kynurenine aminotransferase (KAT) activity. Finally, the ratio kynurenic acid/quinolinic acid was calculated as an index of neuroprotection [14].

### 2.5. Statistical Analysis

Data were reported as median and interquartile range (IQR, for continuous data), or frequency and percentage (categorical data). Among patients, comparisons between two groups were performed using Mann–Whitney test (continuous data) and chi-square test or Fisher’s test (categorical data). In the matched case-control analysis, serum cytokines, FABP2, and tryptophan metabolites were compared between cases and controls or post-infection and non-post-infection cases using Quade’s rank analysis of covariance with BMI, sex, and age as covariates. Correlation between continuous variables was assessed using Spearman rank correlation coefficient. Adjustment for multiple testing was not performed due to the exploratory purpose of this study. All tests were two-sided, and a *p*-value less than 0.05 was considered significant. Statistical analysis was performed using R 4.1 (R Foundation for Statistical Computing, Vienna, Austria) and SPSS 27 (Chicago, IL, USA).

## 3. Results

### 3.1. Patients

The analysis included 40 patients with ME/CFS (14 males and 26 females, median age 33 years, disease duration 6 years) (Table 1). Thirty-one patients had already received a diagnosis of ME/CFS from other colleagues. For nine patients, ME/CFS was a new diagnosis.

Nine patients had severe ME/CFS with a Bell score ≤ 25%. The other patients had a serious disease with a Bell score of 26–65% (Table 1).

ME/CFS started with an infection in 19 patients (47.5%). Infections mostly regarded the upper airways. Three patients developed ME/CFS after Lyme disease and one patient after acute giardiasis during a trip to a tropical country.

All patients fulfilled the criteria for the diagnosis of ME/CFS; some of them, however, presented peculiarities that could not be discounted. These rare manifestations included differences in the way ME/CFS started, the presence of symptoms suggestive of immune disease (dry eyes, oral aphthae), neurological anomalies (jerks, paresthesias, hypersomnia), signs suggestive of autonomic dysregulation (livedo reticularis, acrocyanosis) and hyperlaxity (Table 2). Some patients presented anomalies at the muscle biopsy that could not be attributed to known diseases (Table 2). Finally, some presented genetic abnormalities whose importance is unclear (Table 2).

### 3.2. Follow-Up

Median follow-up was 41 months (IQR 32–47) (Table 3). Most patients (57%) reported worsening of symptoms during follow-up, with fatigue (49%) and cognitive problems (28%) as the prevailing symptom (Table 3). For 18% of patients’ symptoms remained unchanged. Twenty-two percent of patients reported improvement. No differences were found during follow-up between patients starting or not starting with an infection.

Two of the patients who improved (17 and 21 years old, both females, disease duration 1 and 8 years) had recurrent bouts of fever, muscle pain, and flu-like symptoms. During such periods, serum levels of C reactive protein (CRP) and amyloid A increased slightly. Patients also had modest increases of IL-4, TNF-α, IL-17A, and IFN-γ. IL-1β, IL-18, and IL-33 were within normal limits or undetectable, and no mutations were found in genes related to the pathogenesis of autoinflammatory syndromes (MEFV, MVK, TNFRSF1A, NLRP3, and NLRP12). One patient responded rapidly to colchicine, the Bell scale grading increasing from 30 to 100% in a few days. The other, who responded partially to colchicine, was treated with monthly canakinumab (IL-1β antagonist), with dramatic results (Bell scale grading went from 20 to 90% within hours after the first dose, post-exertional malaise and brain fog disappeared, and orthostatic intolerance improved markedly). Since no alternative diagnosis emerged, we have retained these patients in the ME/CFS cohort. Both patients have continued treatment, and the improvement is still persisting after 2 years.

### 3.3. Case-Control Study

#### 3.3.1. Intestinal Permeability

**Figure 2:**
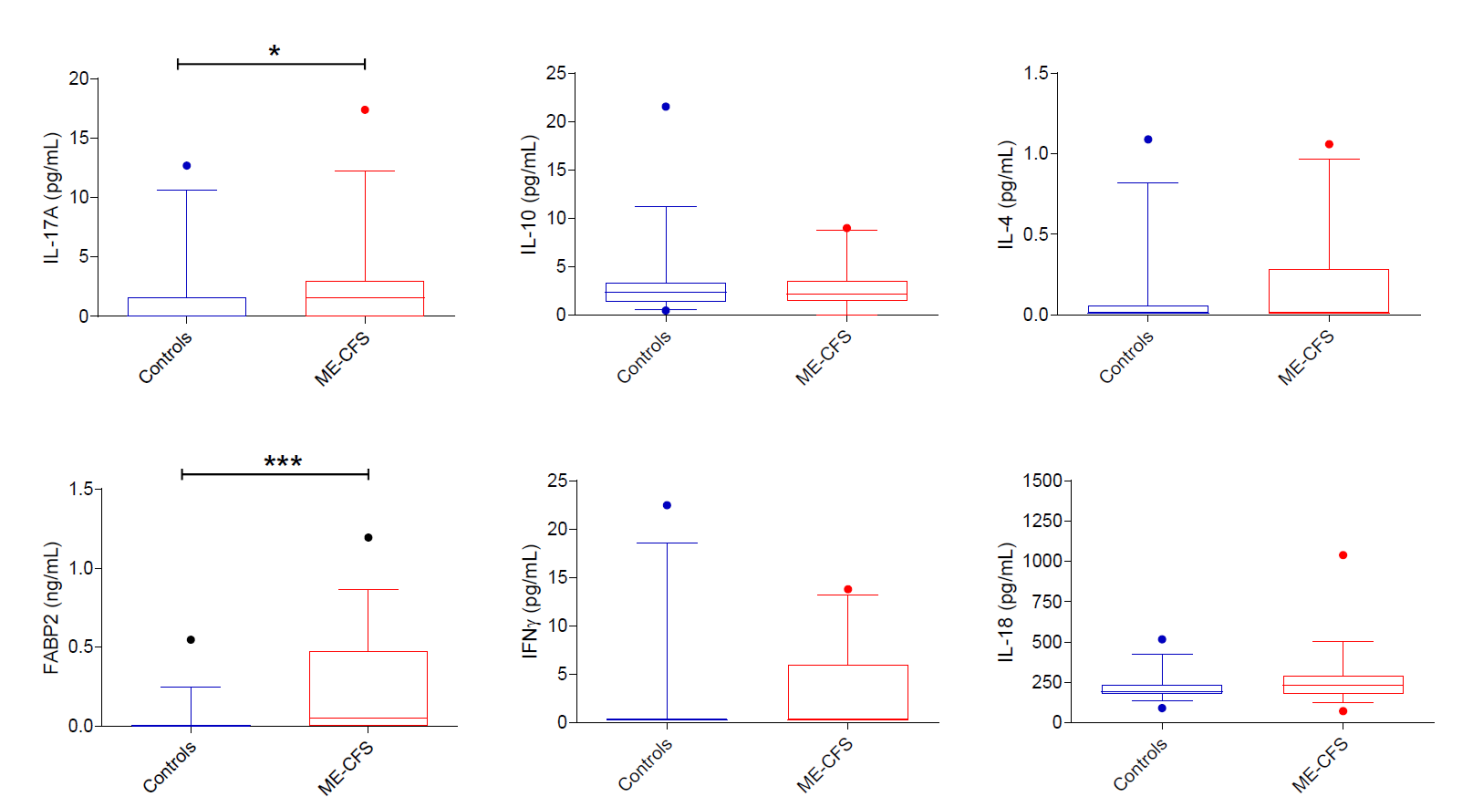
Case-control comparison of serum cytokines and FABP2 (*n* = 9). Serum IL-17A and FABP2 levels are higher in ME/CFS patients than in controls. Data are presented as boxplots with median and interquartile ranges and 5–95 percentiles. Dots represent data outside the 5–95 percentiles. * *p* < 0.05 and *** *p* < 0.001; Quade’s rank analysis of covariance with BMI, sex, and age as covariates.

In the case-control analysis (Figure 2), serum levels of FABP-2 were significantly higher in ME/CFS patients than in controls (F_1,72_ = 24.022, *p* < 0.001, η_p_^2^ = 0.255). No correlation was found between FABP-2 levels and bowl habits or intestinal complaints.

#### 3.3.2. Cytokines

Thirty-nine patients had baseline measurements of several serum cytokines. In most cases, cytokine concentrations were within normal limits. The cytokines more frequently increased were TGF-β, IL-2, and TNF-α (Table 2).

In the case-control analysis (Figure 2), serum levels of IL-17 were higher in ME/CFS patients than in controls (F_1,73_ = 5.901, *p* = 0.018, η_p_^2^ = 0.075), while no differences between ME-CFS patients and controls was found concerning IL-4, IL-10, IL-18, and IFN-γ. In both ME/CFS patients and controls, serum cytokines were not correlated with clinical characteristics nor with any of the other variables measured in this study (data not shown).

#### 3.3.3. Tryptophan Metabolites

**Figure 3:**
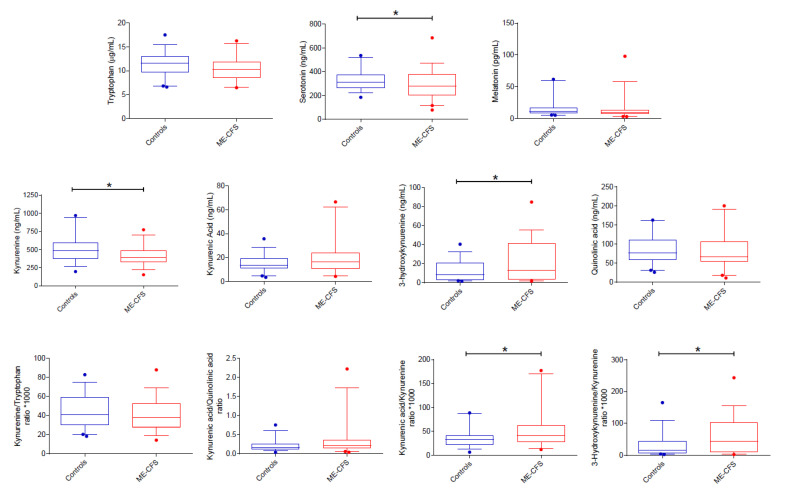
Case-control comparison of tryptophan metabolites via serotonin and kynurenine (*n* = 39). Serum levels of serotonin and kynurenine are lower and those of 3-hydroxykynurenine higher in ME-CFS patients than in controls. The kynurenic acid/kynurenine and 3-hydroxykynurenine/kynurenine ratios, indirect measures of kynurenine aminotransferases and kynurenine 3-monooxygenase enzymatic activities, respectively, are higher in ME-CFS patients than in controls. Data are presented as boxplots with median and interquartile ranges and 5–95 percentiles. Dots represent data outside the 5–95 percentiles. * *p* < 0.05; Quade’s rank analysis of covariance with BMI, sex, and age as covariates.

As shown in Figure 1, tryptophan can be metabolized through the kynurenine (95%) and the serotonin pathways (2%). With respect to controls, ME/CFS patients had differences in the serum concentration of metabolites pertaining to both pathways when controlling for the possible confounding effect of age, sex, and BMI (Figure 3).

Serum levels of tryptophan tended to be lower in ME/CFS patients than in controls (F_1,75_ = 3.979, *p* = 0.05, η_p_^2^ = 0.050). Concerning the serotonin pathway, serum levels of serotonin were lower in ME/CFS patients (F_1,75_ = 4.169, *p* = 0.045, η_p_^2^ = 0.053), while no difference was seen for melatonin (Figure 3). Concerning the kynurenine pathway, ME/CFS patients had lower serum levels of kynurenine (F_1,75_ = 6.657, *p* = 0.012, η_p_^2^ = 0.082) and higher levels of 3-hydroxy-kynurenine (F_1,74_ = 4.499, *p* = 0.037, η_p_^2^ = 0.057) (Figure 3). No differences were seen for quinolinic and kynurenic acids.

Considering the kynurenine/tryptophan ratio, no difference was found between ME/CFS patients and controls. In contrast, patients with ME/CFS had higher 3-hydroxykynurenine/kynurenine (F_1,75_ = 6.025, *p* = 0.016, η_p_^2^ = 0.074) and kynurenic acid/kynurenine (F_1,75_ = 6.072, *p* = 0.016, η_p_^2^ = 0.075) ratios (Figure 3).

No differences were observed between ME/CFS patients and controls for the neuroprotective ratio kynurenic acid/quinolinic acid (Figure 3; F_1,75_ = 2.967, *p* = 0.089, η_p_^2^ = 0.038).

Mode of symptom onset (infectious vs. non-infectious), the evolution of the clinical picture (improved, unchanged, worsened), symptom frequency, and intensity did not correlate with any of the measured biomarkers (data not shown).

### 3.4. Comparisons between Post-Infectious and Non-Post-Infectious ME/CFS Patients

Patients with post-infectious ME/CFS reported more frequently a fast start (*p* < 0.0001) and a higher Bell score (*p* = 0.02), and a tendency to be older (*p* = 0.05) than patients with non-post-infection ME/CFS (Table 4). Starting with or without infection was not associated with differences in symptom scores, bowel habit, abdominal pain, the diagnosis of irritable bowel syndrome, or with the presence of CFS/fatigue/fibromyalgia or immune diseases in the family (Table 4). Concerning circulating levels of cytokines, FABP-2 and tryptophan metabolites, after controlling for sex, age, and BMI, we found a tendency to lower serum levels of IL-18 (*p* = 0.068), lower kynurenine (*p* = 0.026), and lower kynurenine/tryptophan ratio (*p* = 0.015) in post-infection than in non-post-infection ME/CFS patients (Table 5). No differences between the two groups were seen for the other cytokines, FABP-2, and other tryptophan metabolites (Table 5).

## 4. Discussion

Our results indicate that ME/CFS patients differ from control healthy subjects for several serum biomarkers, including FABP-2, IL-17A, and tryptophan metabolites, such as kynurenine, serotonin, 3-hydroxykynurenine, and the ratios kynurenic acid/quinolinic acid and 3-hydroxykynurenine/kynurenine. Moreover, a difference in serum levels of kynurenine and the ratio of kynurenine/tryptophan is also present within ME/CFS patients according to whether the disease started after an infection or not.

### 4.1. Study Population

Our patients fulfilled the criteria of the Canadian Consensus Criteria for the diagnosis of ME/CFS, and for the majority of them, the diagnosis of ME/CFS was also made by independent colleagues. It is clear, however, that they were a heterogeneous group of individuals with differences in disease start, symptoms, laboratory data, and genetic background. Some of the rarer aspects of our patients, such as jerks, hyperlaxity, and livedo reticularis, are long known [29,30], while others, including disturbances in glycogen metabolism, have just been reported [31]. Some clinical and basal laboratory characteristics of our cohort, however, seem novel. Two patients had bouts of low-grade fever and increased serum levels of CRP and amyloid A, as found in auto-inflammatory syndromes [32]. We thus decided to subject them to a trial of drugs used in autoinflammatory syndromes, such as colchicine or the anti-IL1-β monoclonal antibody canakinumab [33], with lasting benefit. Auto-inflammatory syndromes are due to the inappropriate activation of the inflammasome with the production of cytokines of the IL-1β family that mostly act locally so that their serum levels usually remain within normal limits [32]. Although fever and inflammation of the skin, mucosae, serosal surfaces, and osteoarticular structures are cardinal manifestations of these syndromes, fatigue is emerging as an important symptom [34]. We could not attribute these cases to a defined autoinflammatory syndrome, but they clearly responded to the abovementioned treatments, suggesting that it might be convenient to add auto-inflammatory syndromes to the list of differential diagnoses when considering patients with suspected ME/CFS.

One of our patients had a micro-duplication on chromosome 15 (15q13.3), a rare chromosomal disorder that may present with developmental delay, behavioral and psychiatric abnormalities, feeding problems, sleep disturbances, decreased muscle tone, and seizures [35]. The duplication involves the locus of the gene CHRNA7, which codes for the α7 nicotinic acetylcholine receptor (a member of the cholinergic anti-inflammatory pathway) and of the gene CHRFAM7A that acts as a dominant negative inhibitor of the CHRNA7 gene [36,37,38]. At present, we do not know the significance of this anomaly for our patients.

We conclude that our patients had differences in genetic background, disease start, symptoms, basal laboratory data, response to treatment, and evolution.

### 4.2. Intestinal Permeability

ME/CFS patients had increased serum levels of FABP-2, a low molecular weight protein involved in the intracellular traffic of fatty acids, which comprises 4–6% of enterocyte cytosolic proteins, is undetectable or present in very low concentrations in the serum of healthy persons and is used as a marker of increased intestinal permeability [39]. Our observation partially agrees with evidence obtained by Giloteaux et al. [5], who found in a larger cohort of patients increased serum levels of Lipopolysaccharide Binding Protein, another index of increased intestinal permeability, while FABP-2 was increased but not significantly.

The mechanism leading to increased intestinal permeability remains unclear. Intestinal complaints are frequent among patients with ME/CFS, and a recent paper examining fecal bacterial metagenomics in a cohort of patients with ME/CFS concluded that IBS comorbidity was the strongest factor driving separation of data into topological networks [40].

Components of the normal intestinal flora regulate the barrier function of the intestine and exert anti-infective and anti-inflammatory activity [41]. An imbalance in the composition of the intestinal microbiome, well documented in patients with ME/CFS [5,6], could thus contribute to the genesis of the intestinal barrier dysfunction.

### 4.3. Cytokines

In agreement with the literature, no specific cytokine profile could be identified in our patients [2,42].

In the case-control study, the only cytokine with a serum concentration different from controls was IL-17A, which was modestly but significantly increased in ME/CFS patients.

IL-17A is a cytokine produced by multiple cell types (CD4^+^ T cells, CD8^+^ T cells, γδ T cells, invariant natural T cells, innate lymphoid cells, and lung memory T lymphocytes) that can be either pro-inflammatory (in the skin) or protective (in the airways and the intestine) [43]. In the intestine, IL-17A regulates the formation of tight junctions, increases the release of secretory IgA, and favors the production of antibacterial peptides; thus, its increase in our patients might represent a response to a local noxa [43]. On the other side, Th17 polarization and increased production of IL-17A have recently been linked to an increased extracellular concentration of lactate [44]. Considering that in patients with ME/CFS, the intestinal microbiome has an increased ability to produce lactate [45], and that tissue hypoperfusion due to anomalies of peripheral blood flow auto-regulation has been proposed as a possible pathogenetic mechanism of ME/CFS [22], the relationship between energy metabolism and Th17 polarization may merit further scrutiny. Finally, it has been recently suggested that IL-17A may play a role in depression associated with psoriasis and obesity [46]. The present data suggest that it might be worthwhile to see if a relationship exists between IL-17A serum levels and ME/CFS-associated depression.

### 4.4. Tryptophan and Kynurenine Metabolites

Changes in peripheral circulating levels of tryptophan and some of its metabolites via serotonin and kynurenine are considered good biomarkers of their changes in the brain [14,47]. Indeed, most of the brain kynurenine is derived from tryptophan outside the central nervous system. Kynurenine then crosses the blood-brain barrier and is metabolized to kynurenic acid, xanthurenic acid, or 3-hydroxykynurenine [14,48]. 3-hydroxykynurenine is further metabolized to quinolinic acid (Figure 1). Interestingly, among kynurenine metabolites, 3-hydroxykynurenine crosses the blood–brain barrier readily, while kynurenic acid and quinolinic acid, due to high polarity, do not [48].

Metabolites of the kynurenine pathway play a significant role in the homeostasis of the central nervous system, playing both neuroprotective and neurotoxic effects [14,49]. In fact, kynurenine exerts anti-inflammatory activity by binding to aryl hydrocarbon receptor and stimulating the production of regulatory T cells [50]. On the other hand, 3-hydroxykynurenine is neurotoxic since it undergoes oxidation in physiological conditions, producing highly reactive hydroxyl radicals [51]. Kynurenic acid, on the opposite, is neuro-protective since it inhibits all excitatory amino acid receptors (NMDA, kainate, AMPA), inhibits the 7α acetylcholine receptor, binds to aryl hydrocarbon receptor and G-protein coupled receptor 35 (GPR35), inhibits the circuit IL-23/IL-17, and acts as an oxy-radical scavenger [14,48,52,53]. Quinolinic acid is neurotoxic since it stimulates NMDA receptors in specific brain regions, favors lipid peroxidation, inhibits gluconeogenesis, and inhibits mitochondrial monoamine oxidase activity [48].

In ME/CFS, blood tryptophan concentration has been found increased by some authors [15] and unchanged by others [16,54]. In our cohort of patients, serum tryptophan concentration was not different from controls, although patients tended to present lower values (*p* = 0.05). Patients had, instead, lower levels of kynurenine, higher levels of 3-hydroxykynurenine, and an increase in the ratios kynurenic acid/kynurenine (indirect index of KAT activity) and 3-hydroxykynurenine/kynurenine (indirect index of KMO activity).

The mechanisms leading to low serum levels of kynurenine remain undefined, the change being compatible with the decreased transformation of tryptophan into kynurenine and/or, more likely, the increased transformation of kynurenine into kynurenic acid and 3-hydroxykynurenine as we found an increase in the ratios of kynurenic acid/kynurenine and 3-hydroxykynurenine/kynurenine. It is also unclear why patients with a post-infectious start had lower levels of kynurenine than patients not starting with an infection.

In ME/CFS patients, we found increased serum levels of 3-hydroxykynurenine but normal levels of its product quinolinic acid. Since 3-hydroxykynurenine crosses the blood–brain barrier freely [48], increased blood levels of 3-hydroxykynurenine could translate into increased transfer of this toxic molecule to the brain.

Our findings differ from those of Groven et al. [54], who, in a group of patients with ME/CFS, found decreased plasma levels of anthranilic acid, a neuroprotective kynurenine derivative not considered in this study, and a decrease in the ratio kynurenic acid/quinolinic acid, while tryptophan, kynurenine, kynurenic acid, and 3-hydroxykynurenine were not different from controls. On the other side, our data agree in part with evidence recently presented by Hoel et al. [22], who reported lower levels of kynurenine and kynurenic acid and normal levels of tryptophan and serotonin in ME/CFS patients. These discrepancies could be due to differences between the populations studied, given the high heterogeneity within ME/CFS patients. For this reason, we here also included a nosological description of our study population so that it could be used for comparisons with future studies in the field. A further and important point that may explain possible discrepancies between our and the abovementioned studies is the fact that serum samples in Groven et al. [54] and Hoel et al. [22] were collected without restrictions about the feeding state, which is an important factor in determining circulating levels of tryptophan and its metabolites [14]. Of interest, changes in tryptophan biomarkers similar to those found in our patients (low tryptophan, kynurenine, and serotonin and high 3-hydroxykynurenine) have been observed in diabetic ketoacidosis [55,56]. Thus, it is possible that part of the discrepancy between our findings and those of Groven et al. [54] and Hoel et al. [22] may rely on a particular response to fasting by patients with ME/CFS, who present an increased expression of enzymes involved in ketone body metabolism [57] and, for their energy needs, may depend on fatty acid β–oxidation more than healthy controls [8]).

In ME/CFS patients, we also found low serum levels of serotonin, which is over 90% produced by intestinal enterochromaffin cells and is mostly associated with platelet granules [14]. Our findings bear similarities with the observation that circulating serotonin is low in patients with irritable bowel syndrome with constipation [58], a condition characterized by low serotonin concentration in the intestinal wall [59].

### 4.5. Relevance of the Present Findings for the Explanation of ME/CFS Pathogenesis

Overall, we found no significant correlation among cytokines, FABP2, and tryptophan metabolites, likely indicating that inflammation, anomalies of the intestinal barrier, and changes of tryptophan metabolism may be independently associated with the establishment of disease. Despite clear differences among patients, however, our findings remain compatible with the view that persons with ME/CFS may have common or largely shared pathogenetic mechanisms. Indeed, in the present study, changes in tryptophan metabolites and increased IL-17A levels are both compatible with anomalies in the sphere of energy metabolism.

### 4.6. Limitations

This study has several limitations: first, the small sample size; second, its essentially exploratory nature; third, the use of ELISA methods for cytokine measurement that were not of the highest sensitivity, an aspect important for cytokines normally present in blood at very low concentrations; fourth, the lack of deep knowledge about patient diet, supplements, vitamins, and probiotics could have influenced the gut status and tryptophan metabolism; fifth, uncertainty about the extent to which serum levels of tryptophan metabolites reflect changes occurring in the central nervous system; sixth, the presence of control subjects with serum levels of some biomarkers outside the 5–95 percentile (see Figure 2 and Figure 3) is possibly due to undiagnosed underlying disease; however, even if these potential outliers were excluded, the differences observed in the case-control analyses would remain significant; seventh, we did not investigate the physical activity of healthy controls, a factor known to influence serum levels of inflammatory cytokines and tryptophan metabolites [60]. Therefore, the possible confounding effect of exercise in the case-control study was not considered.

## 5. Conclusions

We found substantial heterogeneity among patients with ME/CFS; however, in spite of many differences, patients shared traits of possible significance for the explanation of their symptoms. Our data suggest that clinical aspects and serum biomarkers related to inflammation, intestinal function, and tryptophan metabolism deserve to be further investigated, both for the identification of ME/CFS subtypes and as a way towards a personalized patient care approach. This implies, on one side, the use of larger patient samples and, on the other side, the use of all modern medical tools for the diagnosis and treatment of individual cases.

## Figures and Tables

**Table 1:** ME/CFS patient characteristics.

Variable	All (*n* = 40)
Age, years ^a^	32 (23–46)
Males	14 (35%)
Disease duration, years ^a,b^	6 (3–12)
Symptom start:	
Slow (months)	18/40 (45%)
Fast (weeks)	22/40 (55%)
ME/CFS, fatigue, or fibromyalgia in the family	13/38 ^d^ (34%)
Immune diseases in the family	17/38 ^d^ (44%)
Symptom score (0–6): ^a^	
Fatigue	6 (5–6)
PEM	5 (5–6)
Unrefreshing sleep	4 (4–5)
Pain	4 (3–5)
Cognitive anomalies	5 (4–5)
OI/POTS	4 (4–5)
GI abnormalities	4 (3–4)
Flu-like symptoms	4 (3–5)
Bell scale score (0–100%) ^a,c^	30 (25–50)
Bowel habit: ^a^	
Normal	23/38 ^d^ (61%)
Styptic	10/38 ^d^ (26%)
Diarrheic	5/38 ^d^ (13%)
Abdominal pain—IBS ^e^	23/40 (58%)

Data expressed as *n* (%) or ^a^ median (IQR). PEM—post-exertional malaise; OI—orthostatic intolerance; POTS: postural orthostatic tachycardia syndrome; GI—gastro-intestine; IBS—irritable bowel syndrome. Data were not available in ^b^ 1, ^c^ 4, and ^d^ 2 patients; ^e^ diagnosed according to the Rome IV criteria [28].

**Table 2:** Symptoms, laboratory data, and genetic abnormalities present in a minority of patients.

Variable	Number of Patients (%)	Notes
Disease start		
After a neoplasia	2 (5)	Meningioma, Hodgkin lymphoma
After a vaccine	1 (2)	
Symptoms, signs, and laboratory data		
Skin hyperlaxity	4 (10)	
Livedo reticularis	2 (5)	
Dry eyes/mouth	2 (5)	
Oral aphthae	2 (5)	
Paresthesias	3 (7)	
Jerks	2 (5)	
Hypersomnia	2 (5)	
Bouts of fever, flu-like symptoms, increase in CRP and serum amyloid A	2 (5)	Response to colchicine, canakinumab
Muscle biopsy anomalies	1 (2)	Thickened basal membrane of small vessels, glycogen accumulation within myocytes
Serum cytokines above normal *		
IL-1α	2 (5)	
IL-2	9 (22)	
IL-6	2 (5)	
IL-8	1 (2)	
TNF-α	7 (17)	
TGF-β	13 (32)	
Genetic abnormalities		
CASQ1 gene mutation	1 (2)	N227I, heterozygous. Muscle biopsy: thickened vessel basal membrane, fibrils of unknown nature around myocytes.
15q13.3 duplication (340 BP)	1 (2)	
Ehlers–Danlos syndrome	1 (2)	
Myotonia congenita	1 (2)	

BP—base pairs; CRP—C reactive protein; * measured in 39 patients.

**Table 3:** Follow-up clinical information of the 40 ME/CFS patients.

Follow-Up, Months ^a^	41 (32–47)
Symptom evolution during follow up:	
Unchanged	8/40 (20%)
Worsened	21/40 (52%)
Improved	11/40 (28%)
Prevailing symptoms during follow-up:	
Fatigue	20 (50%)
PEM	7 (17%)
Unrefreshing sleep	2 (5%)
Pain	6 (15%)
Cognitive anomalies	12 (30%)
OI/POTS	1 (2%)
Abdominal pain/IBS ^b^	1 (2%)
Flu-like	1 (2%)

Data expressed as *n* (%) or ^a^ median (IQR). PEM—post-exertional malaise; OI—orthostatic intolerance; POTS—postural orthostatic tachycardia syndrome; IBS—irritable bowel syndrome. ^b^: diagnosed according to the Rome IV criteria [28].

**Table 4:** Clinical characteristics of post-infectious (*n* = 19) and non-post-infections (*n* = 21) ME/CFS patients.

Variable	ME/CFS Did Not Start after an Infection (*n* = 21)	ME/CFS Started after an Infection (*n* = 19)	Statistics
Age, years ^a^	30 (20–44)	35 (28–48)	U = 261.5, *p* = 0.093
Males	8 (38%)	6 (32%)	χ^2^ = 0.186, *p* = 0.66
Disease duration, years ^a,b^	8 (3–13)	5 (3–11)	U = 169.5, *p* = 0.59
Symptom start:			χ^2^ = 23.089, *p* < 0.0001
Slow (months)	17/21 (81%)	1/19 (5%)	
Fast (weeks)	4/21 (19%)	18/19 (95%)	
ME/CFS, fatigue or fibromyalgia in the family	7/19 ^d^ (37%)	6/19 (31%)	χ^2^ = 0.000, 0.99
Immune diseases in the family	9/19 ^d^ (47%)	8/19 (42%)	χ^2^ = 0.010, 0.92
Symptom score (0–6): ^a^			
Fatigue	6 (5–6)	6 (5–6)	
PEM	5 (5–6)	5 (5–6)	U = 193.0, *p* = 0.84 U = 211.0
Unrefreshing sleep	5 (4–5)	4 (4–5)	*p* = 0.74 U = 214.0,
Pain	4 (3–5)	4 (3–6)	*p* = 0.69 U = 212.0,
Cognitive anomalies	5 (4–5)	5 (4–5)	*p* = 0.73 U = 179.0
OI/POTS	4 (4–5)	4 (4–5)	*p* = 0.55 U = 249.0,
GI abnormalities	4 (0–4)	4 (3–5)	*p* = 0.17 U = 250.0,
Flu-like symptoms	4 (3–4)	5 (3–5)	*p* = 0.16 U = 242.0, *p* = 0.24
Bell scale score (0–100%) ^a,c^	30 (22–41)	43 (30–50)	U = 236.0, *p* = 0.02
Bowel habit: ^a^			χ^2^ = 5.353, *p* = 0.07
Normal ^d^	11/20 (55%)	12/18 (67%)	
Styptic ^d^	8/20 (40%)	2/18 (11%)	
Diarrheic ^d^	1/20 (5%)	4/18 (22%)	
Abdominal pain—IBS ^e^	13/21 (62%)	10/19 (53%)	χ^2^ = 0.351, *p* = 0.55

Data expressed as *n* (%) or ^a^ median (IQR). PEM—post-exertional malaise; OI—orthostatic intolerance; POTS—postural orthostatic tachycardia syndrome; GI—gastro-intestine; IBS—irritable bowel syndrome. Data were not available in ^b^ 1, ^c^ 4, and ^d^ 2 patients; ^e^ diagnosed according to the Rome IV criteria [28].

**Table 5:** Serum levels of cytokines, FABP-2, and tryptophan metabolites along the serotonin and kynurenine pathways in post-infection (*n* = 19) and non-post-infection (*n* = 21) ME/CFS patients.

	ME/CFS Did Not Start after an Infection (*n* = 21)	ME/CFS Started after an Infection (*n* = 19)	Statistics
Cytokines			
IL-17A (pg/mL)	1.6 (0.2–5.3)	1.6 (0.0–2.8)	F_1,36_ = 0.073, *p* = 0.789, η_p_^2^ = 0.002
IL-10 (pg/mL)	2.2 (1.7–4.2)	2.0 (1.3–2.7)	F_1,36_ = 1.311, *p* = 0.260, η_p_^2^ = 0.035
IL-4 (pg/mL)	0.0 (0.0–0.5)	0.0 (0.0–0.3)	F_1,36_ = 0.503, *p* = 0.485, η_p_^2^ = 0.021
IFN-γ (pg/mL)	0.3 (0.3–8.2)	0.3 (0.3–4.5)	F_1,36_ = 0.205, *p* = 0.654 η_p_^2^ = 0.006
IL-18 (pg/mL)	255.3 (195.9–303.4)	196.9 (155.6–250.1)	F_1,36_ = 4.521, *p* = 0.068, η_p_^2^ = 0.091
FABP-2 (ng/mL)	0.0 (0.0–0.5)	0.13 (0.0–0.5)	F_1,34_ = 0.492 *p* = 0.488, η_p_^2^ = 0.015
Kynurenine pathway			
Tryptophan (μg/mL)	9.36 (8.26–12.01)	10.68 (9.56–11.99)	F_1,37_ = 1.640, *p* = 0.208, η_p_^2^ = 0.042
Kynurenine (ng/mL)	413.4 (339.2–539.6)	347.6 (260.1–397.9)	F_1,37_ = 5.410, *p* = 0.026, η_p_^2^ = 0.128
3-hydroxykynurenine (ng/mL)	12.3 (3.3–41.7)	17.5 (5.9–39.6)	F_1,37_ = 1.102, *p* = 0.301, η_p_^2^ = 0.029
Kynurenic acid (ng/mL)	16.6 (11.0–24.1)	14.2 (9.1–35.8)	F_1,37_ = 0.216, *p* = 0.645, η_p_^2^ = 0.006
Quinolinic acid (ng/mL)	59.6 (40.9–102.6)	67.2 (59.6–111.6)	F_1,37_ = 0.298, *p* = 0.588, η_p_^2^ = 0.008
Kynurenine/tryptophan ratio ∗ 1000	44.1 (32.9–57.9)	33.2 (24.8–38.8)	F_1,37_ = 6.525, *p* = 0.015, η_p_^2^ = 0.150
3-hydroxykynurenine/kynurenine ratio ∗ 1000	42.4 (7.8–97.9)	80.6 (19.0–107.7)	F_1,37_ = 0.499, *p* = 0.485, η_p_^2^ = 0.013
Kynurenic acid/kynurenine ratio	39.6 (26.5–59.7)	50.6 (27.6–84.1)	F_1,37_ = 0.313, *p* = 0.579, η_p_^2^ = 0.008
Kynurenic acid/quinolinic acid ratio	0.2 (0.1–0.4)	0.2 (0.1–0.3)	F_1,37_ = 0.131, *p* = 0.719, η_p_^2^ = 0.004
Serotonin pathway			
Serotonin (ng/mL)	301.4 (218.3–382.4)	258.8 (180.4–377.4)	F_1,37_ = 1.261, *p* = 0.269, η_p_^2^ = 0.033
Melatonin (pg/mL)	9.8 (6.3–20.2)	10.2 (8.1–13.7)	F_1,37_ = 0.076, *p* = 0.784, η_p_^2^ = 0.002

Data are median (IQR). Comparisons have been computed using Quade’s rank analysis of covariance with BMI, sex, and age as covariates.

## Data Availability

The data sets analyzed during the current study are available from the corresponding author on reasonable request.

## Supplementary Materials

The following are available online at https://www.mdpi.com/article/10.3390/biomedicines9111724/s1, Table S1: Study design.
