# Supplementary material for: Tryptophan Metabolites, Cytokines, and Fatty Acid Binding Protein 2 in Myalgic Encephalomyelitis/Chronic Fatigue Syndrome
Source: Biomedicines. 2021 Nov 19;9(11):1724. doi: 10.3390/biomedicines9111724 (PMC8615774; doi:10.3390/biomedicines9111724)
Supplement: Supplementary file 1 [file biomedicines-09-01724-s001.zip › biomedicines-1445078-supplementary.pdf]

Table S1. Study design.

**Phase**	**Timing**	**Actions Taken**
Recruitment	January 1, 2017-June 31, 2018	70 patients with unexplained fatigue were visited and 45 were diagnosed with ME/CFS using the CCC Criteria. Out of 45 patients with ME/CFS, 40 accepted to take part to the study
Case-control study	July 1, 2018-December 1, 2019	After an overnight fast, blood was collected from 40 ME/CFS patients and from 40 age- and sex-matched healthy controls. On frozen serum samples the following variables were measured: IL-4, IL-10, IL-17A, IL-18, IFN-γ; Fatty Acid Binding Protein 2: tryptophan, kynurenine, 3-hydroxykynurenine, kynurenic acid, quinolinic acid, serotonin and melatonin.
Follow up	December 2, 2019- May 31, 2021	Patients were contacted periodically by phone due to the COVID-19 emergency. During May 2021 patients responded to a written questionnaire reviewing family history, drugs taken, ME/CFS symptoms (categorized according to the CCC criteria), any new symptom, impression about the evolution of the disease. Patients were encouraged to contact the investigators (A.B. and C. Z.) by phone or E mail about points of uncertainty or needeing comments.
